# The Photocatalytic Application of Semiconductor Stibnite Nanostructure Synthesized via a Simple Microwave-Assisted Approach in Propylene Glycol for Degradation of Dye Pollutants and its Optical Property

**DOI:** 10.1186/s11671-017-2356-6

**Published:** 2017-11-09

**Authors:** Eksuree Saksornchai, Jutarat Kavinchan, Somchai Thongtem, Titipun Thongtem

**Affiliations:** 1 School of Science, University of Phayao, Phayao, 56000 Thailand; 2 Department of Physics and Materials Science, Faculty of Science, Chiang Mai University, Chiang Mai, 50200 Thailand; 3 Center of Excellence in Materials Science and Technology, Chiang Mai University, Chiang Mai, 50200 Thailand; 4 Department of Chemistry, Faculty of Science, Chiang Mai University, Chiang Mai, 50200 Thailand

**Keywords:** Microwave-assisted synthesis, Sb_2_S_3_ nanostructure, Energy gap, Photocatalytic activity

## Abstract

Stibnite (Sb_2_S_3_) semiconducting material was successfully synthesized by a rapid and facile microwave route using antimony chloride (SbCl_3_) and sodiumthiosulfate (Na_2_S_2_O_3_) dissolved in propylene glycol (PG) containing different hydroxyethyl cellulose (HEC) masses. The phase identification, morphology, and elemental composition of products were characterized by X-ray diffraction (XRD), transmission electron microscopy (TEM), field- emission scanning electron microscopy (FE-SEM), energy dispersive X-ray (EDX) spectroscopy, and Fourier transform infrared spectroscopy (FTIR). The results revealed the orthorhombic phase of Sb_2_S_3_ single crystal-forming sheaf-like nanostructure, and a possible formation mechanism was proposed and discussed. Its direct band gap calculated from UV-visible absorption is 1.60 eV. In this research, the photocatalytic activities of Sb_2_S_3_ nanostructure were investigated through the degradation of methyl orange (MO) and methylene blue (MB) under visible light irradiation. The as-obtained 0.30 g HEC-added solution (0.3 HEC-Sb_2_S_3_) photocatalyst exhibited better photocatalytic activity than the other products, which degraded 91% of MO within 300 min and 90% of MB within 240 min under the Xe-lamp irradiation. The first-order plot was fitted with this experiment which the rate constant (*k*) of 0.3 HEC-Sb_2_S_3_ for MO and MB degradation are 0.0085 and 0.0098 min^−1^, respectively. Therefore, the new experience with a novel and simple synthetic procedure of Sb_2_S_3_ photocatalyst that exhibits the characteristics of a highly effective photocatalyst under visible light irradiation was discovered.

## Background

Presently, the photocatalytic activity of semiconductor nanomaterial has drawn considerable interest worldwide because they have significant potential for the degradation of dye pollutants in wastewater and elimination of environmental contaminations. A serious problem with toxic synthetic dyes was arisen from discharging the wastes of textile, paper, and plastic industries. Consequently, it is necessary to treat these dye pollutants in water before draining to natural resources.

Typically, a variety of procedures have been used for removal of synthetic dyes from water, including membrane separation, microbiological decomposition, adsorption, and photocatalysis [1–7]. Among them, the most potential method with a high efficiency is using photocatalytic material for dye degradation [8]. Over the last 10 years, semiconductor photocatalyst has grown significantly. A primary focus on TiO_2_ has been applied to environmental problems, due to it has traditionally formed as photocatalyst under irradiation for pollutant degradation by generating strong oxidants such as HO^•^ radicals that can rapidly and non-selectively degrade organic compounds [9]. Other types of semiconductors such as ZnO, α-Fe_2_O_3_, CdS, ZnS, ZnO-SnO_2,_ CeO_2_-TiO_2_, and Ag_3_PO_4_ composite were also used as photocatalyst because of their optimum optoelectrical, physical, and chemical properties [10]. A variety of approaches have been developed to prepare the unique structure of semiconductor nanostructures such as one-dimensional nanostructures due to their low number of grain boundaries, rapid charge transfer dynamics, and high specific area can improve the photocatalytic performance [9, 11–15]. In recent years, there are a number of processes used to synthesize photocatalysts with different morphologies: hydrothermal routes to synthesize ZnO-SnO_2_ hollow spheres [16], α-Fe_2_O_3_ hollow spheres [17], α-Fe_2_O_3_ nanospindles, nanotubes, and nanotires [18], and CeO_2_-TiO_2_ nanobelt heterostructures [14]; microwave-assisted sol-gel method to synthesize N-Cu-activated carbon (AC)/TiO_2_ nanoparticles [11] and carbon-based (N,Fe)-codoped TiO_2_ nanoparticles [12]; and a combined chemical vapor deposition (CVD) technique and hydrothermal route to prepare Ag_3_PO_4_/CNFs/silica-fiber hybrid composites [15].

Previously, the pure TiO_2_ photocatalyst can only absorb ultraviolet light, due to its large band gap (3.0–3.2 eV) [14]. To solve these problems, numerous strategies have been studied to reduce the charge recombination and enhance visible light utilization of photocatalysts. Chalcogenide semiconductor photocatalyst is one of the promising materials that has been found to mark enhancement of the photocatalytic activity in the visible region because of its narrow band gap [19–22].

Recently, chalcogenide photocatalysts can play an important role in the degradation of dyes, such as In_2_S_3_ used for MB photocatalytic degradation under visible irradiation [9]; SnS, Sn_2_S_3_, and SnS_2_ quantum dots were studied for the photocatalytic degradation of MB [20]; the photocatalysis of Sb_2_S_3_ nanowires was investigated for the degradation of MO [21]; SnS microstructure was used for the photocatalytic degradation of Rhodamine B (RhB) under sunlight [22].

Herein, semiconducting photocatalysts of main group metal chalcogenide A_2_X_3_ (A = As, Sb, Bi; X = S, Se, Te), such as Sb_2_S_3_ or stibnite, have been studied intensively due to their numerous applications including optoelectronic, thermo-electric technologies, solar energy utilization, and semiconducting photocatalyst [23–25]. Different approaches were used for synthesizing of Sb_2_S_3_: refluxing [23], spray pyrolysis [24], hydrothermal process [25], and microwave-assisted synthesis [26]. In this research, microwave-assisted synthesis in a facile solution-phase method was used for synthesizing Sb_2_S_3_. This method has a lot of benefits: very simple, effective, low cost, fast, and precise control of the reaction (mixing condition and size and shape controlling) [11–13, 27]. The results show that this method has high potential and reproducibility. Thus, it is one of the most promising techniques used for controlling the morphology of products. In addition, the photocatalytic activities were also evaluated through the degradation of methyl orange (MO) solution and methylene blue (MB) solution under visible light irradiation.

## Experimental

### Preparation of Sb_2_S_3_ Photocatalysts

All chemicals used in this experiment were analytical grade and used without further purification. To synthesize Sb_2_S_3_, each of 2 mmol of antimony chloride (SbCl_3_, assay: 99.0%, Sigma-Aldrich) and 3 mmol of sodium thiosulfate (Na_2_S_2_O_3,_ ≥ 99.0%, Sigma-Aldrich) was dissolved in 30 mL propylene glycol (PG, assay: 99.5%, QRëC). They were thoroughly mixed, followed by pH adjusting to 4 using 0.1 M HCl (diluted from concentrated HCl (37% *v*/*v*), Sigma-Aldrich), and stirred for 15 min. Then, 0.10, 0.20, and 0.30 g of HEC (M_w_ ~ 250,000, Sigma-Aldrich) were added to the mixed solutions and irradiated with microwave radiation at 300 W for 20 min to form 0.1 HEC-Sb_2_S_3_, 0.2 HEC-Sb_2_S_3_, and 0.3 HEC-Sb_2_S_3_ products, respectively. The as-obtained product appears as black precipitates which were separated by filtration, washed with absolute ethanol, and dried at the 70 °C for 24 h, for further characterization. For the present synthesis, different contents of hydroxyethyl cellulose (HEC) were also used as a template.

### Characterization of Photocatalysts

Phase and purity were characterized by an X-ray diffractometer (XRD, SIEMENS D500) operating at 20 kV and 15 mA and using Cu-K_α_ line in 2θ range of 10–60 deg. Shape, size, and composition of the precipitates were investigated by a field-emission scanning electron microscope (FE-SEM, JEOL JSM-6335F) equipped with an energy dispersive X-ray (EDX) analyzer operating at 15 kV, a transmission electron microscope (TEM, JEOL JEM-2010), a selected area electron diffractometer (SAED) operating at 200 kV, and Fourier transform infrared spectroscopy (FTIR). The optical property was studied by a UV-visible spectrometer (Lambda 25, PerkinElmer) using a UV lamp with a resolution of 1 nm.

### Measurement of Photocatalytic Activity

The photocatalytic activities of Sb_2_S_3_ nanostructure were evaluated by MO (C_14_H_14_N_3_NaO_3_S) and MB (C_16_H_18_ClN_3_S) degradation under visible light irradiation. A 300-W Xe lamp equipped with a 420-nm cut-off filter was used as a visible light source. Practically, 30 mg of the photocatalyst was dispersed in a 100 mL of 11 mg L^−1^ MO solution and 100 mL of 10 mg L^−1^ MB solution, respectively. Prior to irradiation, the dye solution was continuously stirred for 1 h in the dark for adsorption-desorption equilibrium. Then, the visible light was turned on to start photocatalysis. During the process, 3 mL of suspension was withdrawn every a certain time interval and centrifuged to precipitate the photocatalyst. The absorbance of the clear solution or the change of MO and MB concentration were determined by UV-visible spectrophotometer (PerkinElmer, Lambda 25) using the maximum absorbance at 464 and 665 nm, respectively. The photocatalytic efficiency of MO and MB degradation was calculated using the formula

1 $$ \mathrm{Degradation}\  \mathrm{efficiency}=\frac{C_0-C}{C_0}\times 100\%, $$

where *C*_0_ is the initial concentration of MO or MB concentration and *C* is the concentration of MO or MB after visible light irradiation within the elapsed time (*t*).

## Results and Discussion

### Phase and Morphology Analysis

**Fig. 1:**
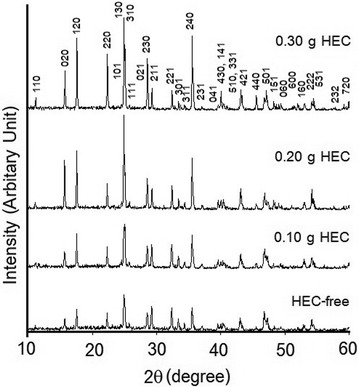
XRD spectra of Sb_2_S_3_ synthesized in the solution containing 0, 0.10, 0.20, and 0.30 g HEC

The crystalline degree and phase purity of Sb_2_S_3_ crystal were shown in Fig. 1 which have been characterized by an X-ray diffractometer (XRD). At 300 W of microwave power, the XRD results revealed the pure phase of Sb_2_S_3_ with Pbnm space group, in accordance with the JCPDS File no. 06-0474 [28]. All the diffraction peaks can be readily indexed to an orthorhombic phase of stibnite. Upon increasing the mass of HEC, the XRD peak turned into sharper and higher intensity, implying that the crystalline degree was improved—in accordance with the good order of the lattice atom reported by Thongtem et al. [29]. In this research, the highest content of HEC-added solution was 0.30 g due to the problem in the synthesis of Sb_2_S_3_ in PG is very viscous, and the delay of kinetic control can cause agglomeration due to inefficient physical mixing [27].

**Fig. 2:**
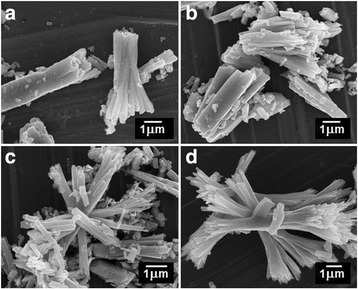
FE-SEM images of **a** Sb_2_S_3_, **b** 0.1 HEC-Sb_2_S_3_, **c** 0.2 HEC-Sb_2_S_3_, and **d** 0.3 HEC-Sb_2_S_3_ products

To reveal the shape and size of the as-obtained Sb_2_S_3_, Fig. 2 is a set of SEM images which show the development of Sb_2_S_3_ morphologies in different contents of HEC-added solutions. There are a number of nanorods with irregular shape in the HEC-free solution. When 0.10 g HEC was added to the mixed solution, it caused each nanorod to split more to be a bundle of nanorods. A bundle of nanorod was enlarged and more open at their end tips in 0.20 g HEC-added solution. Finally, the Sb_2_S_3_ morphology was changed into a sheaf-like structure and more beautiful in 0.30 g HEC-added solution. The present morphology is similar to the report by Ota et al. [25] who started the straw tide-like morphology with 18-h reaction time synthesized by hydrothermal. Previously, the straw-bundled-like nanorods were synthesized in 1,4-butanediol using polyvinylpyrrolidone (PVP) as surfactant via refluxing polyol process for 30 min as reported by Zhang et al. [23]. But for this work, the sheaf-like structures were successfully synthesized by microwave-assisted synthesis for 20 min, spends less reaction time than the previous researches.

**Fig. 3:**
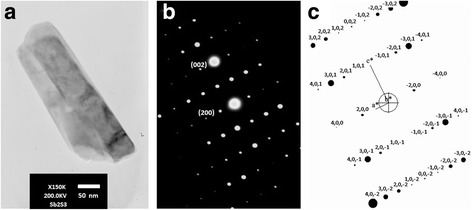
**a** TEM image, **b** SAED pattern, and **c** simulated pattern of the 0.3 HEC-Sb_2_S_3_ product

TEM image, SAED, and simulated patterns [30] of Sb_2_S_3_ synthesized in 0.30 g HEC-added solution (0.3 HEC-Sb_2_S_3_) are illustrated in Fig. 3. Each individual nanorod growing along the [001] direction [30]—in accordance with the growth direction identified by Ota et al. [25], Kavinchan et al. [26], and Wang et al. [31]. The SAED pattern shows a systematic array of white spots of orthorhombic phase of Sb_2_S_3_ single crystal, corresponding to the diffraction pattern obtained by simulation [30].

**Fig. 4:**
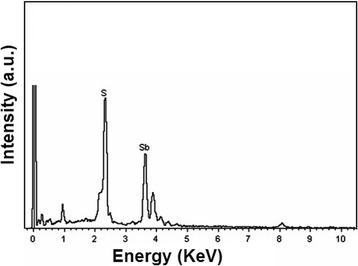
EDX spectrum of 0.3 HEC-Sb_2_S_3_ product

Quantification of the EDX spectrum (Fig. 4) of 0.3 HEC-Sb_2_S_3_ product provides close to 2:3 atomic ratio of Sb:S—in good accordance with the stoichiometric composition of Sb_2_S_3_. It should be noted that other peaks were also detected—caused by the electronic transition of copper stub and carbon tape used for holding the analyzed sample [32].

### Energy Gap Analysis

**Fig. 5:**
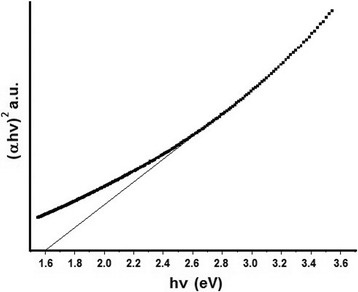
The (*αhν*)^2^ and *hν* plot of 0.3 HEC-Sb_2_S_3_ product

Figure 5 shows the photonic absorbance attenuating through the Sb_2_S_3_ nanorods. The absorption was controlled by two photon energy (*hν*) ranges, corresponding to the high and low energies. When photon energy is greater than the energy band gap (*E*_*g*_), the absorption was linearly increased with the increase in photon energy. This research shows the *(αhν)* ^2^ and (*hν*) plot for direct allowed transition, where *α* is the total absorption coefficient, *h* is the Plank constant, and *ν* is the photonic frequency [33]. The band gap energy of Sb_2_S_3_ is therefore estimated to be 1.60 eV, in accordance with 1.59–1.60 eV of Sb_2_S_3_ determined by Alonso et al. [34] compare to those products reported by other researchers, such as the 1.52 eV prepared by Zhu et al. [35], 2.08 eV by Ota et al. [25], and 2.27 eV by Chate et al. [36]. The band gap of this report is less than 1.80 eV of flower-like and 2.08 eV of Sb_2_S_3_ nanorods determined by Ota et al. [25] and 2.27 eV of thin film Sb_2_S_3_ calculated by Chate et al. [36]. This analysis implies that the Sb_2_S_3_ nanostructure has the potential to absorb visible light and can be used for solar-light-driven applications and optical nanodevices [22, 35, 37].

### A Possible Mechanism of Sb_2_S_3_ Formation and Simulation

A possible formation mechanism of Sb_2_S_3_ was controlled by hydrolytic decomposition of sodiumthiosulfate as a sulfur source. First, antimony (III) ions (Sb^3+^) in the solution formed complexes with thiosulfate. In the mixed solution containing Sb^3+^ and S^2−^ at pH 4 as an acidic medium, thiosulfate ions slowly released sulfide ions to combine with antimony (III) ions, and colloidal complexes formed. Finally, the black precipitates of Sb_2_S_3_ were synthesized. Moreover, the concentration of Sb^3+^ and S^2−^ ions in the solution also played the role in the formation rate of Sb_2_S_3_ [36, 38].

2 $$ 2{\mathrm{S}\mathrm{b}}^{3+}+3{\mathrm{S}}_2{{{\mathrm{O}}_3}^2}^{-}\to {\mathrm{S}\mathrm{b}}_2{\left({\mathrm{S}}_2{\mathrm{O}}_3\right)}_3 $$

3 $$ {\mathrm{S}}_2{{{\mathrm{O}}_3}^2}^{-}+{\mathrm{H}}^{+}\to \mathrm{S}+{{\mathrm{H}\mathrm{SO}}_3}^{-} $$

4 $$ \mathrm{S}+2{\mathrm{e}}^{-}\to {\mathrm{S}}^{2-} $$

5 $$ 2{\mathrm{S}\mathrm{b}}^{3+}+3{\mathrm{S}}^{2-}\to \kern0.5em {\mathrm{S}\mathrm{b}}_2{\mathrm{S}}_3 $$

**Fig. 6:**
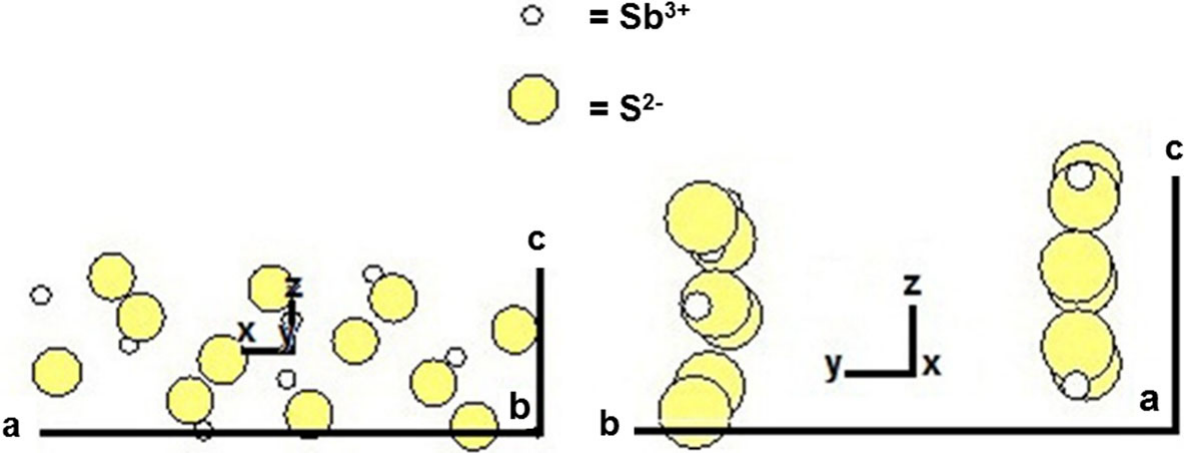
The growth of Sb_2_S_3_ obtained by computer modeling

Figure 6 illustrates Sb_2_S_3_ structure obtained by simulation. It shows that the infinite chains of stoichiometric composition of atoms run normal to the axis. The binding force of the chain in b direction is greatly weaker than the one along the chain (*a*- and *c*-axis) [29, 39]. The cleavage can easily occur in the (010) plane—caused by the binding between these chains—and is considerably weaker than that within the chains. Thus, the growth direction is preferential along the [001] direction [39]. The crystal splitting is also associated with fast crystal growth, due to the super saturation that exceeds a certain critical value [26, 39, 40]. In this work, HEC was used as a soft template and capping agent—composed of long macromolecules with a number of hydroxyl groups. The chain molecules were entangled nuclei in liquid medium to form very tiny particles with active site on the surfaces. Consequently, the crystals grew out of active sites and HEC molecules adsorbed on the side walls of the crystals [39]—resulting the products to become lengthy and more beautiful in 0.30 g HEC-added solution. The crystal splitting is also associated with the sudden crystal growth, due to the saturated solution exceeding a certain critical value [39, 40]. Comparing the previous researches, nanorods synthesized using sodium dodecyl sulfate (SDS) as a surfactant by Ota et al. [25], the double flower-like microcrystal using dodecyltrimethylammonium bromide (DTAB) as a surfactant by Wu et al. [41], the dumb-bells using polyvinylpyrrolidone as a template by Kavinchan et al. [26], and nanowires using polyethylene glycol with molecular weight of 6 kDa (PEG-6000) by Wang et al. [31], the present sheaf-like structures synthesized using HEC as capping agent are more beautiful—affected by the stability and reliability of using polymers as templates [29].

### Photocatalytic Activity

**Fig. 7:**
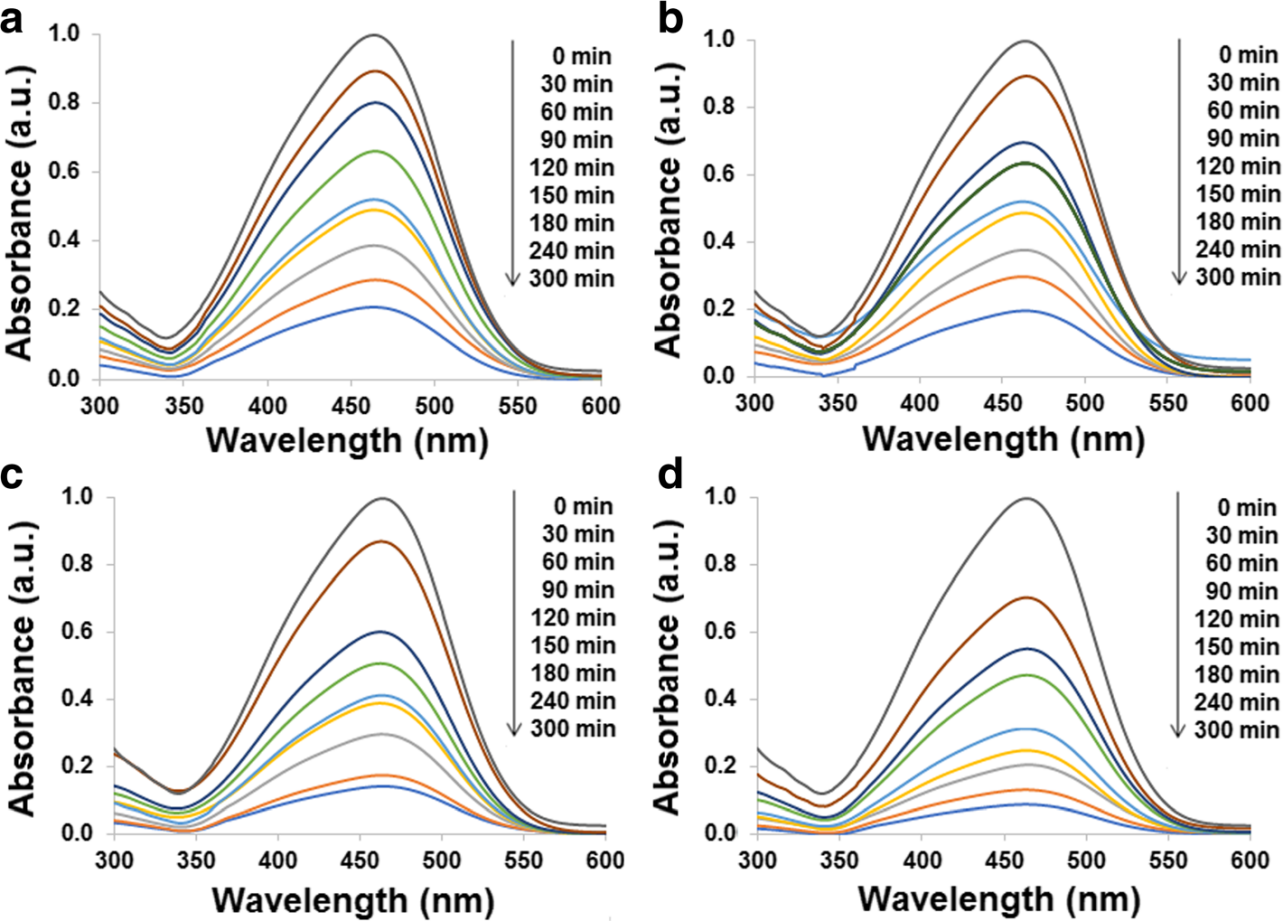
UV-visible spectra of MO solution using **a** Sb_2_S_3_, **b** 0.1 HEC-Sb_2_S_3_, **c** 0.2 HEC-Sb_2_S_3_, and **d** 0.3 HEC-Sb_2_S_3_ as photocatalytic materials

**Fig. 8:**
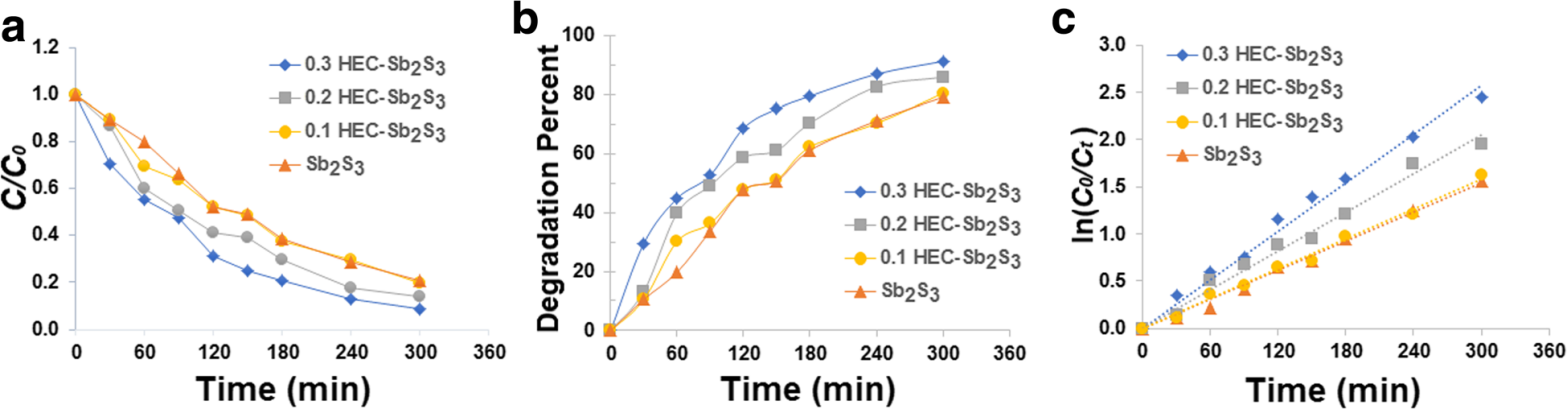
**a**–**c** The adsorption kinetic, photocatalytic degradation, and first-order plot of MO solution by Sb_2_S_3_, 0.1 HEC-Sb_2_S_3_, 0.2 HEC-Sb_2_S_3_, and 0.3 HEC-Sb_2_S3 photocatalysts

Figure 7 shows the absorption spectra of MO solution using Sb_2_S_3_, 0.1 HEC-Sb_2_S_3_, 0.2 HEC-Sb_2_S_3_, and 0.3 HEC-Sb_2_S_3_ products (Fig. 7a–d) as photocatalyst, respectively. The degradation efficiency of MO solution (Fig. 8a, b) was 79% within 300 min by using Sb_2_S_3_ whereas the degradation efficiency of MO was gradually increased, which correspond to 80, 86, and 91% at the same time by using 0.1 HEC-Sb_2_S_3_, 0.2 HEC-Sb_2_S_3_, and 0.3 HEC-Sb_2_S_3_ photocatalysts, respectively—influenced by the more uniform as-prepared product with good crystallinity and the large surface area [42]. Compared to the MO degradation efficiency (71% within 150 min) of Sb_2_S_3_ nanowires reported by Zhang et al. [21], the MO degradation efficiency of this work (75% within 150 min) is more. The first-order plot was fitted with this experiment, and rate constant of MO degradation was obtained by the following equation

6 $$ \ln \left({C}_{\mathrm{o}}/{C}_t\right)= kt, $$

where *C*_o_ is the initial concentration at time *t* = 0, *C* is the concentration at time *t*, *k* is the first-order rate constant, and *t* is the irradiation time. As can be observed in Fig. 8c, the computed rate constants for Sb_2_S_3_, 0.1 HEC-Sb_2_S_3_, 0.2 HEC-Sb_2_S_3_, and 0.3 HEC-Sb_2_S_3_ photocatalysts were gradually increased which were 0.0052, 0.0053, 0.0068, and 0.0085 min^−1^, respectively for the MO degradation.

**Fig. 9:**
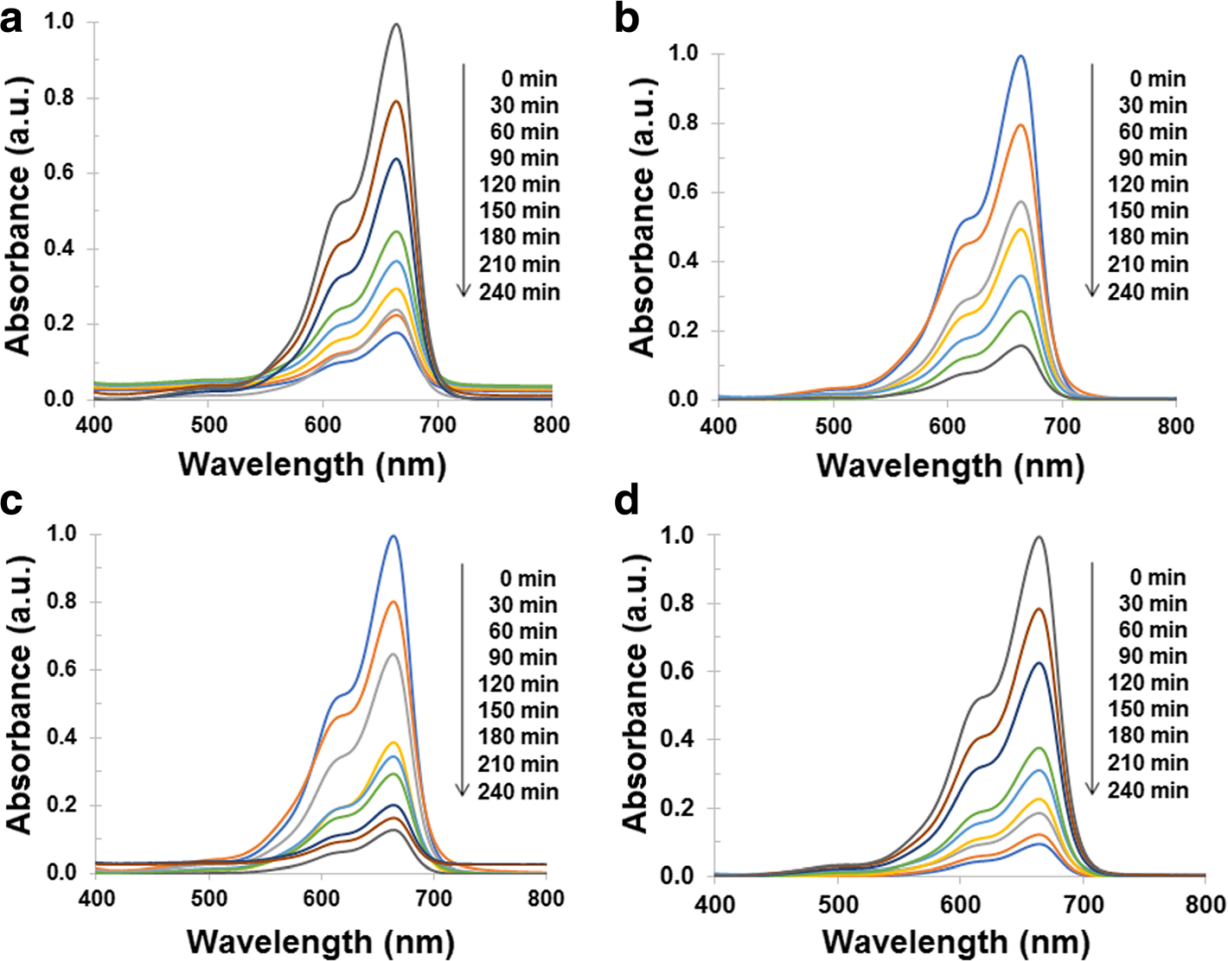
UV-visible spectra of MB solution using **a** Sb_2_S_3_, **b** 0.1 HEC-Sb_2_S_3_, **c** 0.2 HEC-Sb_2_S_3_, and **d** 0.3 HEC-Sb_2_S_3_ as photocatalytic materials

**Fig. 10:**
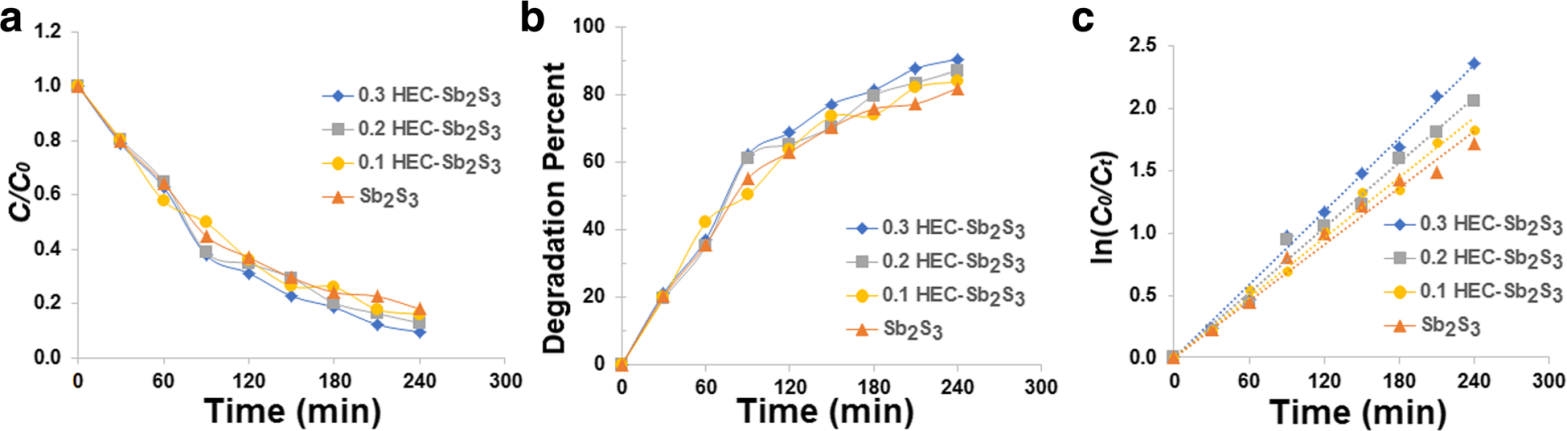
**a**–**c** The adsorption kinetic, photocatalytic degradation, and first-order plot of MB solution by Sb_2_S_3_, 0.1 HEC-Sb_2_S_3_, 0.2 HEC-Sb_2_S_3_, and 0.3 HEC-Sb_2_S3 photocatalysts

The absorption spectra of MB solution using Sb_2_S_3_, 0.1 HEC-Sb_2_S_3_, 0.2 HEC-Sb_2_S_3_, and 0.3 HEC-Sb_2_S_3_ as photocatalyst were shown in Fig. 9. The photocatalytic activities and the first-order plot of MB degradation are shown in Fig. 10. The degradation efficiency of MB solution was 82% by using Sb_2_S_3_, compared to using 0.1 Sb_2_S_3_-HEC, 0.2 Sb_2_S_3_-HEC, and 0.3 Sb_2_S_3_-HEC as photocatalyst, the degradation percent were respectively calculated to be 84, 87, and 90% within 240 min. The results show that photocatalytic activities of 0.3 HEC-Sb_2_S_3_ have the highest degradation efficiency of all samples in both of MO and MB solution, due to its large specific surface area of nanorods that can promote the electron-hole separation on surface area and increase the photocatalytic reaction site [14, 42]. The calculated first-order rate constants (Fig. 10c) were 0.0076, 0.0080, 0.0087, and 0.0098 min^−1^ for Sb_2_S_3_, 0.1 HEC-Sb_2_S_3_, 0.2 HEC-Sb_2_S_3_, and 0.3 HEC-Sb_2_S_3_, respectively. These results indicate that 0.3 HEC-Sb_2_S_3_ photocatalyst was more efficient in MB degradation than MO degradation—due to an equal degradation efficiency close to 90% on MB and MO but the MB degradation time less than MO solution. The results reveal the rate constant (*k*) of 0.3 HEC-Sb_2_S_3_ for MO (0.0085 min^−1^) and MB (0.0098 min^−1^) solution were higher than the other samples—caused by the good crystallinity and the large surface area of the product [21, 42].

**Fig. 11:**
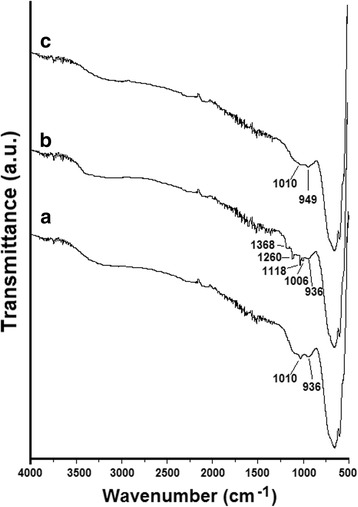
FTIR spectra of **a** 0.3 Sb_2_S_3_-HEC, **b** 0.3 Sb_2_S_3_-HEC adsorbed by MO, and **c** 0.3 Sb_2_S_3_-HEC adsorbed by MB, respectively

Figure 11 shows the FTIR spectra in the range of 500–4000 cm^−1^. As can be seen in Fig. 11a, the peaks at 1010, 949, and 599 cm^−1^ correspond to the bonds of metallic compounds and M–OH modes of product [43–45]. The absorption peaks around 3400 and 1600 cm^−1^ related to the stretching and bending vibration of O–H bond, respectively [44–46]. The peaks between 1100 and 1500 cm^−1^ are in accordance with the C–H stretching band. Figure 11b shows the peaks at 1368, 1260, 1118, and 1006 cm^−1^ of 0.3 HEC-Sb_2_S_3_ adsorbed by MO sample were attributed to the C–N vibrations, C–N aromatic stretching vibrations, the vibration of C–N bonds, and C–H in-plane bending vibration of benzene rings of MO molecules, respectively [9, 14, 47]. Figure 11c shows the peak at 1010 cm^−1^ of 0.3 HEC-Sb_2_S_3_ adsorbed by MB was broadened compare to 0.3 HEC-Sb_2_S_3_—caused by the bonding with 0.3 HEC-Sb_2_S_3_ and MB molecules [45, 46]. Moreover, the rather strong peak at 949 cm^−1^ can be attributed to modes form as a result of H-bond of the type N_het ···_ HO (stretching heterocycle_···_ HO) [48]. These were obtained from hydrogen bonds between the heterocyclic N atoms of MB molecules and water H of Sb_2_S_3_ surfaces [46, 48]. These results indicated that MO molecules act as the anion in aqueous solution may be absorbed on the surface of photocatalyst—causing the peaks of MO molecules on FTIR spectra. On the other hand, MB act as the cation in the aqueous solution that can be interacted with the hydroxyl (–OH) group on the surface area of the photocatalyst—causing the peak at 1010 cm^−1^ to decrease after adsorption [43–48].

### Possible Photocatalytic Mechanism

**Fig. 12:**
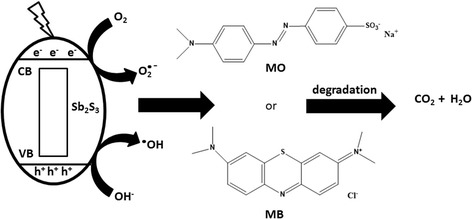
Schematic diagram of a possible process of visible-light-driven photodegradation of MO and MB by Sb_2_S_3_

Figure 12 shows the MO and MB degradation mechanism induced by Sb_2_S_3_ nanostructure. During the photocatalytic process, electrons and holes reacted with the adsorbed substances (O_2_ and OH^−^) on the surfaces of crystals to form oxidative radicals such as O_2_^•−^ and ^•^OH [49–51] when irradiation energy is equal to or higher than its energy band gap (1.60 eV for 0.3 HEC-Sb_2_S_3_) [21, 51]. Then, the MO and MB organic molecules were destroyed and efficiently degraded to form H_2_O and CO_2_ molecules as the final products [10, 52–54].

### Conclusions

In summary, a sheaf-like Sb_2_S_3_ nanostructure composed of a number of nanorods act as the visible-light-driven photocatalyst was successfully synthesized by a simple microwave radiation. The purpose of this work was to gain new experience with a simple synthetic procedure of Sb_2_S_3_ photocatalyst that has a high degradation efficiency. The first-order plot was fitted with this experiment. The as-obtained 0.3 HEC-Sb_2_S_3_ photocatalyst exhibited better photocatalytic activity than the other products, which degraded 91% of MO within 300 min and 90% of MB within 240 min under the Xe-lamp irradiation. The 0.3 HEC-Sb_2_S_3_ product is beneficial to the photodegradation of the MO and MB dye. It may be mainly attributed to its band gap (*E*_g_ = 1.60 eV), which cover range of the solar spectrum and has strong absorption of the visible light. Consequently, these results suggest that the as-prepared 0.3 HEC-Sb_2_S_3_ product exhibits a good photocatalytic activity under the visible light irradiation and it is one of the promising photocatalytic materials for wastewater treatment.

## Abbreviations

CVD: Chemical vapor deposition
DTAB: Dodecyltrimethylammonium bromide
EDX: Energy dispersive X-ray
FE-SEM: Field-emission scanning electron microscopy
FTIR: Fourier transform infrared spectroscopy
HEC: Hydroxyethyl cellulose
MB: Methylene blue
MO: Methyl orange
PG: Propylene glycol
PVP: Polyvinylpyrrolidone
Rh B: Rhodamine B
SAED: Selected area electron diffraction
SDS: Sodium dodecyl sulfate
TEM: Transmission electron microscopy
XRD: X-ray diffraction

## References

[1] Wanga J, Qin L, Lin J, Zhu J, Zhang Y, Liu J, Bruggen BV (2017). Enzymatic construction of antibacterial ultrathin membranes for dyes removal. Chem Eng J.

[2] Solís M, Solís A, Pérezb HI, Manjarrez N, Floresa M (2012). Microbial decolouration of azo dyes: a review. Process Biochem.

[3] Qi Y, Yang M, Xu W, He S, Men Y (2017). Natural polysaccharides-modified graphene oxide for adsorption of organic dyes from aqueous solutions. J Colloid Interface Sci.

[4] Jiang Q, Lu Y, Huang Z, Hu J (2017). Facile solvent-thermal synthesis of ultrathin MoSe_2_ nanosheets forhydrogen evolution and organic dyes adsorption. Appl Surf Sci.

[5] Farhadi S, Dusek M, Siadatnasab F, Eigner V, Mokhtari A (2017). First organic–inorganic hybrid nanomaterial constructed from a Keggin-type polyoxometallate and a copper-dithiocarbamate complex: sonochemical synthesis, crystal structure and its adsorption performance for organic dye pollutants. Polyhedron.

[6] Zhang K, Chen W, Wang Y, Li J, Chen H, Gong Z, Chang S, Ye F, Wang T, Chu W, Zou C, Song L (2015). Cube-like Cu_2_MoS_4_ photocatalysts for visible light-driven degradation of methyl orange. AIP Adv.

[7] Omidvar A, Jaleh B, Nasrollahzadeh M (2017). Preparation of the GO/Pd nanocomposite and its application for the degradation of organic dyes in water. J Colloid Interface Sci.

[8] Paola D, Lópeza EG, Marcìa G, Palmisano L (2012). A survey of photocatalytic materials for environmental remediation. J Hazard Mater.

[9] Shen T, Wang C, Sun J, Jiang C, Wang X, Li X (2015). TiO_2_ modified abiotic-biotic process for the degradation of azo dye methyl orange. RSC Adv.

[10] Hoffmann MR, Martin ST, Choi W, Bahnemannt DW (1995). Environmental applications of semiconductor photocatalysis. Chem Rev.

[11] Tian F, Wu Z, Yan Y, Ye B, Liu D (2016). Synthesis of visible-light-responsive Cu and N-codoped AC/TiO_2_ photocatalyst through microwave irradiation. Nanoscale Res Lett.

[12] Tian F, Wu Z, Tong Y, Wu Z, Cravotto G (2015). Microwave-assisted synthesis of carbon-based (N,Fe)-codoped TiO_2_ for the photocatalytic degradation of formadehyde. Nanoscale Res Lett.

[13] Liu D, Wu Z, Tian F, Ye B, Tong Y (2016). Synthesis of N and La co-doped TiO_2_/AC photocatalyst by microwave irradiation for the photocatalytic degradation of naphthalene. J Alloys Compd.

[14] Tian J, Sang Y, Zhao Z, Zhou W, Wang D, Kang X, Liu H, Wang J, Chen S, Cai H, Huang H (2013) Enhanced photocatalytic performances of CeO_2_/TiO_2_ nanobelt heterostructures. Small. 10.1002/smll.201202346 10.1002/smll.201202346 23681828

[15] Chen P, Wu L, Yao W (2016). Novel synthesis of Ag3PO4/CNFs/silica-fiber hybrid composite as an efficient photocatalyst. J Taiwan Inst Chem E.

[16] Jin C, Ge C, Jian Z, Wei Y (2016). Facile synthesis and high photocatalytic degradation performance of ZnO-SnO_2_ hollow spheres. Nanoscale Res Lett.

[17] Jin C, Ge C, Xu G, Peterson G, Jian Z, Wei Y, Zhu K (2017). Influence of nanoparticle size on ethanol gas sensing performance of mesoporous α-Fe_2_O_3_ hollow spheres. Mater Sci Eng B.

[18] Liu C, Ma J, Chen H (2012). Periodical structure conversion and its mechanism in hematite: from nanospindles, to nanotubes, to nanotires. RSC Adv.

[19] Vallejo W, Díaz-Uribe C, Rios K. Methylene blue photocatalytic degradation under visible irradiation on In_2_S_3_ synthesized by chemical bath deposition. Adv Phys Chem. 2017;Art ID6358601. doi:10.1155/2017/6358601.

[20] Cheraghizade M, Jamali-Sheini F, Yousefi R, Niknia F, Mahmoudian MR, Sookhakian M (2017). The effect of tin sulfide quantum dots size on photoctalytic and photovoltaic performance. Mater Chem Phys.

[21] Zhang H, Hu C, Ding Y, Lin Y (2015). Synthesis of 1D Sb_2_S_3_ nanostructures and its application in visible-light-driven photodegradation for MO. J Alloys Compd.

[22] Kabouche S, Bellel B, Louafi Y, Trari M (2017). Synthesis and semiconducting properties of tin(II) sulfide: application to photocatalytic degradation of Rhodamine B under sun light. Mater Chem Phys.

[23] Zhang R, Chen X, Mo M, Wang Z, Zhang M, Liu X, Qian Y (2004). Morphology-controlled growth of crystalline antimony sulfide via a refluxing polyol process. J Cryst Growth.

[24] Kriisa M, Krunks M, Acik IO, Kärber E, Mikli V (2015). The effect of tartaric acid in the deposition of Sb_2_S_3_ films by chemical spray pyrolysis. Mater Sci Semicond Process.

[25] Ota J, Roy P, Srivastava SK, Nayak BB, Saxena AK (2008). Morphology evolution of Sb_2_S_3_ under hydrothermal conditions: flowerlike structure to nanorods. Cryst Growth Des.

[26] Kavinchan J, Thongtem T, Thongtem S (2010). Cyclic microwave assisted synthesis of Sb_2_S_3_ dumb-bells using polyvinylpyrrolidone as a template and splitting agent. Mater Lett.

[27] Haesuwannakij S, Karuehanon W, Mishra VL, Kitahara H, Sakurai H, Kanaoka S, Aoshima S (2014). Size-controlled preparation of gold nanoclusters stabilized by high-viscosity hydrophilic polymers using a microflow reactor. Monatsh Chem.

[28] Powder Diffract. File. JCPDS-ICDD. 12 Campus Boulevard, Newtown Square. PA 19073-3273. USA; 2001.

[29] Thongtem T, Jattukul S, Pilapong C, Thongtem: Hydroxyethyl cellulose-assisted hydrothermal synthesis of Bi_2_S_3_ urchin-like colonies. Curr Appl Phys 2012;12:23-30

[30] Boudias C, Monceau D, CaRIne Crystallography 3.1. DIVERGENT S.A. Centre de Transfert. 60200 Compiègne. France; 1989–98.

[31] Wang G, Cheung CL (2012). Building crystalline Sb_2_S_3_ nanowire dandelions with multiple crystal splitting motif. Mater Lett.

[32] Chumha N, Thongtem T, Thongtem S, Tantraviwat D, Kittiwachana S, Khowphong S (2016). A single-step method for synthesis of CuInS_2_ nanostructures using cyclic microwave irradiation. Ceram Int.

[33] Speight JG (2005). Lange’s handbook of chemistry.

[34] Alonso CM, Peralta EU, Lerma MS, Sato-Berrú RY, Hernández SM, Hu H (2017). Purity and crystallinity of microwave synthesized antimony sulfide microrods. Mater Chem Phys.

[35] Zhu Q, Gong M, Zhang C, Yong G, Xiang S (2009). Preparation of Sb_2_S_3_ nanomaterials with different morphologies via a refluxing approach. J Cryst Growth.

[36] Chate PA, Lakde SD (2015). Characteristics of Sb_2_S_3_ thin films deposited by a chemical method. Int J Thin Fil SciTec.

[37] Omata T, Nose K, Matsuo SO (2009). Size dependent optical band gap of ternary I-III-VI_2_ semiconductor nanocrystals J. Appl Phys.

[38] Meherzi H, Nasr TB, Kamoun N, Dachraoui M (2010). Structural, morphology and optical properties of chemically deposited Sb_2_S_3_ thin films. Phys B.

[39] Pilapong C, Thongtem T, Thongtem S (2010). Hydrothermal synthesis of double sheaf-like Sb_2_S_3_ using copolymer as a crystal splitting agent. J Alloys Compd.

[40] Tang J, Alivisatos AP (2006). Crystal splitting in the growth of Bi_2_S_3_. Nano Lett.

[41] Wu L, Xu H, Han Q, Wang X (2013). Large-scale synthesis of double cauliflower-like Sb_2_S_3_ microcrystallines by hydrothermal method. J Alloys Compd.

[42] Shuai X, Shen W (2012). A facile chemical conversion synthesis of Sb_2_S_3_ nanotubes and the visible light-driven photocatalytic activities. Nanoscale Res Lett.

[43] Han Q, Sun S, San D, Zhu J, Wang X. Room-temperature synthesis from molecular precursors and photocatalytic activities of ultralong Sb_2_S_3_ nanowires. RSC Adv. 2011;1:1364–9.

[44] Pradhan A, Paul A, Rao GR (2017). Sol-gel-cum-hydrothermal synthesis of mesoporous Co-Fe@Al_2_O_3_-MCM-41 for methylene blue remediation. J Chem Sci.

[45] Sodeifien G, Behnood R (2017). Application of microwave irradiation in preparation and characterization of CuO/Al_2_O_3_ nanocomposite for removing MB dye from aqueous solution. J Photochem Photobiol A.

[46] Ganesh RS, Durgadevi E, Navaneethan M, Sharma SK, Binitha HS, Ponnusamy S, Muthamzhchelven C, Hayakawa Y (2017). Visible light induced photocatalytic degradation of methylene blue and rhodamine B from the catalyst of CdS nanowire. Chem Phys Lett.

[47] Hassani KE, Beakou BH, Kalnina D, Oukani E, Anouar A (2017). Effect of morphological properties of layered double hydroxides on adsorption of azo dye methyl orange: a comparative study. Appl Clay Sci.

[48] Ovchinnikov OV, Evtukhova AN, Kondratenko TS, Smimov MS, Khokhlov VY, Erina OV (2016). Manifestation of intermolecular interactions in FTIR spectra of methylene blue molecules. Vib Spectrosc.

[49] Wang H, Yuan X, Wang H, Chen X, Wu Z, Jiang L, Xiong W, Zeng G (2016). Facile synthesis of Sb_2_S_3_/ultrathin g-C_3_N_4_ sheets heterostructures embedded with g-C_3_N_4_ quantum dots with enhanced NIR-light photocatalytic performance. Appl Catal, B.

[50] Shitole KD, Patil AB, Thakur P (2016). Preparation and characterization of Sb_2_S_3_-TiO_2_ nanocomposite for the enhanced photocatalytic degradation and mineralization of azo dye. Environ Eng Sci.

[51] Xie H, Que W, He Z, Zhong P, Liao Y, Wang G (2013). Preparation and photocatalytic activities of Sb_2_S_3_/TiO_2_ nanotube coaxial heterogeneous structure arrays via an ion exchange adsorption method. J Alloys Compd.

[52] Zhang H, Hu C (2011). Effective solar absorption and radial microchannels of SnO_2_ hierarchical structure for high photocatalytic activity. Catal Commun.

[53] Mehrabian M, Esteki Z (2017). Degradationn of methylene blue by photocatalysis of copper assisted ZnS nanoparticle thin films. Optik.

[54] Gao J, Liu C, Wang F, Jia L, Duan K, Liu T (2017). Facile synthesis of heterostructured WS_2_/Bi_2_MoO_6_ as high-performance visible-light-driven photocatalysts. Nanoscale Res Lett.

